# Zoonotic Source Attribution of *Salmonella enterica* Serotype Typhimurium Using Genomic Surveillance Data, United States

**DOI:** 10.3201/eid2501.180835

**Published:** 2019-01

**Authors:** Shaokang Zhang, Shaoting Li, Weidong Gu, Henk den Bakker, Dave Boxrud, Angie Taylor, Chandler Roe, Elizabeth Driebe, David M. Engelthaler, Marc Allard, Eric Brown, Patrick McDermott, Shaohua Zhao, Beau B. Bruce, Eija Trees, Patricia I. Fields, Xiangyu Deng

**Affiliations:** University of Georgia Center for Food Safety, Griffin, Georgia, USA (S. Zhang, S. Li, H. den Bakker, X. Deng);; Centers for Disease Control and Prevention, Atlanta, Georgia, USA (W. Gu, B.B. Bruce, E. Trees, P.I. Fields);; Minnesota Department of Health, St. Paul, Minnesota, USA (D. Boxrud, A. Taylor);; Translational Genomics Research Institute, Flagstaff, Arizona, USA (C. Roe, E. Driebe, D.M. Engelthaler);; US Food and Drug Administration, College Park, Maryland, USA (M. Allard, E.W. Brown);; US Food and Drug Administration, Laurel, Maryland, USA (P. McDermott, S. Zhao)

**Keywords:** Salmonella, source attribution, Salmonella enterica serotype Typhimurium, population structure, machine learning, whole-genome sequencing, United States, zoonoses, bacteria

## Abstract

Increasingly, routine surveillance and monitoring of foodborne pathogens using whole-genome sequencing is creating opportunities to study foodborne illness epidemiology beyond routine outbreak investigations and case–control studies. Using a global phylogeny of *Salmonella enterica* serotype Typhimurium, we found that major livestock sources of the pathogen in the United States can be predicted through whole-genome sequencing data. Relatively steady rates of sequence divergence in livestock lineages enabled the inference of their recent origins. Elevated accumulation of lineage-specific pseudogenes after divergence from generalist populations and possible metabolic acclimation in a representative swine isolate indicates possible emergence of host adaptation. We developed and retrospectively applied a machine learning Random Forest classifier for genomic source prediction of *Salmonella* Typhimurium that correctly attributed 7 of 8 major zoonotic outbreaks in the United States during 1998–2013. We further identified 50 key genetic features that were sufficient for robust livestock source prediction.

Each year, 9.4 million episodes of foodborne illness occur in the United States (*1*). According to the Centers for Disease Control and Prevention, ≈95% of these infections are sporadic, nonoutbreak cases for which specific food exposures and contamination sources remain difficult to determine. The lack of source information for most foodborne infections substantially challenges understanding of the epidemiology of foodborne illnesses and development of intervention measures for their prevention and mitigation. Routine use of whole-genome sequencing (WGS) for foodborne illness surveillance and pathogen monitoring has created a large and quickly expanding wealth of genomes and associated metadata. Much of these data remain largely untapped beyond routine outbreak investigation. 

*Salmonella enterica* is one of the most prevalent foodborne pathogens worldwide, causing >1 million human cases and an economic burden of $3.7 billion annually in the United States alone (*1*,*2*). *S. enterica* serotype Typhimurium is one of the most prevalent causes of human salmonellosis in many countries, including the United States (*3*). *Salmonella* Typhimurium strains display a broad host range and varying degrees of host adaptation (*4*). Diverse subtypes have caused emerging epidemics in recent decades. First isolated in the early 1980s in the United Kingdom, multidrug-resistant *Salmonella* Typhimurium definitive type 104 spread from cattle to other livestock in the country before its global dissemination during the 1990s (*5*). *Salmonella* Typhimurium sequence type (ST) 313 emerged ≈40–50 years ago in sub-Saharan Africa (*6*); its regional spread was linked to invasive disease symptoms and coincided with an HIV pandemic (*6*,*7*). The high prevalence, diverse reservoirs, and dynamic epidemiology of *Salmonella* Typhimurium have made it a paradigm for studying host specificity (*8*) and zoonotic colonization (*9*). Knowing the contribution of major sources of human illness caused by specific pathogens is critical for identifying, evaluating, and prioritizing public health intervention strategies (*10*). Microbiological source attribution relying on subtyping has shown some promise, such as a source attribution model from Denmark based on *Salmonella* serotyping and phage typing (*11*). However, more recent models continued to use traditional phenotypes for *Salmonella* source attribution (*12*). The usefulness of more discriminating molecular subtyping for *Salmonella* source attribution has not been established (*12*).

We hypothesized that microbiological source attribution could be improved by an evolutionary understanding of pathogen populations and a mechanistic inquiry into their source association. We investigated zoonotic source attribution of *Salmonella* Typhimurium under an extensive phylogenomic framework by including a large collection of isolates from 3 major US laboratory surveillance and monitoring programs. We examined genotypic characteristics and metabolic profiles to assess livestock host adaptation and production environment colonization. Machine learning enabled comprehensive and high-resolution screening for key genetic indicators of source association throughout *Salmonella* Typhimurium genomes.

## Materials and Methods

To study the population structure of *Salmonella* Typhimurium and its monophasic variant (I 4,[5],12:i:-), we first sequenced (n = 127) or collected (n = 1,140) 1,267 *Salmonella* Typhimurium genomes (Appendix 1 Table 1; Appendix 2 section 1), comprising 4 sets of genomes. First, we selected human isolates from outbreak and sporadic cases in the United States during 1949–2014 (n = 127, sequenced for this study) to represent diverse pulsed-field gel electrophoresis patterns (n = 51) and multilocus variable-number tandem-repeat analysis patterns (n = 16, for isolates of known pattern) of *Salmonella* Typhimurium as surveyed by PulseNet USA during 1998–2014 (*13*). Second, we chose genomes in the GenomeTrakr database (*14*) as of September 2015 (n = 907) from food, environmental, and wild and livestock animal sources in the United States, Europe, South America, Asia, and Africa. Third, we included retail meat isolates sampled by the US National Antimicrobial Resistance Monitoring System (n = 157). Finally, we compiled other reported *Salmonella* Typhimurium genomes, including human ST313 isolates from sub-Saharan Africa (n = 76) (*6*). Publicly available genomes were confirmed to be *Salmonella* Typhimurium by SeqSero (*15*).

**Figure 1:**
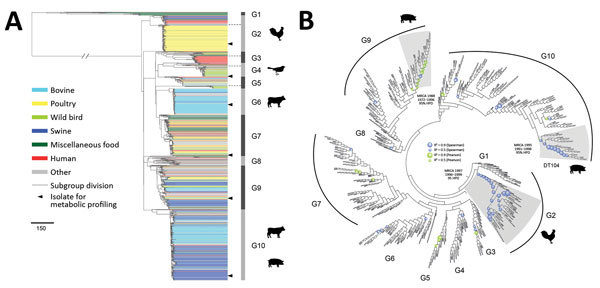
Phylogenetic structure of 1,267 *Salmonella enterica* serotype Typhimurium isolates. A) Maximum-likelihood phylogeny from 46 US states and 39 other countries. The tree was rooted at midpoint. Ten major population groups (G1–G10) were delineated. Each dashed line shows the division of subgroups in G2, G3, G4, and G5 (e.g., G2a and G2b). Each isolate is color coded by source. Arrowheads indicate isolates selected for metabolic profiling using Phenotype Microarrays (Biolog, https://biolog.com). Scale bar indicates number of single-nucleotide polymorphisms. B) Circular cladogram of the same maximum-likelihood phylogeny of the 1,267 isolates. Colored circles indicate internal nodes that had a squared coefficient (R^2^) of the Spearman or Pearson correlation between isolation years and branch lengths >0.4. The sizes of the circle are proportional to the values of R^2^ (0.0–0.9). Clades identified to exhibit temporal signals of single-nucleotide polymorphisms accumulation are shaded in gray. The inferred MRCA age of each clade is shown. HPD, highest posterior density; MRCA, most recent common ancestor.

Overall metabolic potentials of 6 randomly selected representative isolates from 6 major population groups (Figure 1; Appendix 1 Table 2) were evaluated by Phenotype Microarrays (Biolog, https://biolog.com) (Appendix 2 section 8). Principal component analysis was conducted using Phenotype Microarrays results. Phylogenetic analysis, temporal signal screening and most recent common ancestor (MRCA) dating, and putative pseudogene identification were performed (Appendix 2 sections 2–4). A Random Forest classifier (https://www.stat.berkeley.edu/~breiman/randomforest2001.pdf) was built to predict zoonotic sources of *Salmonella* Typhimurium genomes (Appendix 2 sections 5, 6).

## Results

### Population Structure

We constructed a maximum-likelihood phylogeny of the 1,267 isolates based on single-nucleotide polymorphisms (SNPs) identified in the core genome alignments (Figure 1, panel A). We determined 39,562 single-nucleotide variable sites from the alignment. We excluded 154 genome segments potentially involved in recombination from the alignment and SNP identification. The recombinant regions (Appendix 1 Table 3) accounted for 269,366 nt (5.6%) of a typical *Salmonella* Typhimurium genome (4.8 Mb).

Bayesian analysis of the population structure delineated 10 population groups, designated G1–10 (Figure 1, panel A). Most population groups were monophyletic, except G5 and G8. G1 represented a highly divergent population (>9,000 SNPs) from other population groups and contained a high proportion (68.8%) of isolates from seafood, especially from Asia. Human clinical and miscellaneous food isolates (see source classification in Appendix 2, section 5) were widely distributed among population groups; clinical and food isolates were found in every group, except we found no food isolates in G6. Several groups contained major clades associated with particular sources, including G2b with poultry, G4b and G5b with wild birds, G6 with bovine, and G10 with swine and bovine sources (Figure 1, panel A; Appendix 1 Table 4). Other population groups included isolates from diverse sources. G7 accounted for all the aforementioned sources and had more human and food isolates than any other group. Representing 15.4% of the *Salmonella* Typhimurium collection, G7 had 21.6% and 40.2% of all human and food isolates. Eight major foodborne outbreaks during 1998–2012 were included. Of those, 5 were represented by isolates in G7: a 2006 picnic-associated outbreak in the United States, a 2009 multistate peanut butter–associated outbreak in the United States, a 2009 shredded lettuce–associated outbreak in the western United States and Canada, a 2010–2011 multistate alfalfa sprout–associated outbreak in the United States, and a 2012 multistate cantaloupe-associated outbreak in the United States.

To investigate the temporal history of *Salmonella* Typhimurium lineages, we searched temporal signals of SNP accumulation throughout the phylogeny by screening every internal node for strong correlation between isolation years and branch lengths (Figure 1, panel B). Although the entire phylogeny exhibited an overall weak temporal signal (R^2^ <0.2), 3 clades showed moderate temporal signals (R^2^ >0.4), permitting robust age inference of their MRCA. All 3 clades were associated with livestock and originated around the 1990s (Appendix 1 Table 5).

### Differential Abundance of Putative Pseudogenes Among Recently Diverged Clades

**Figure 2:**
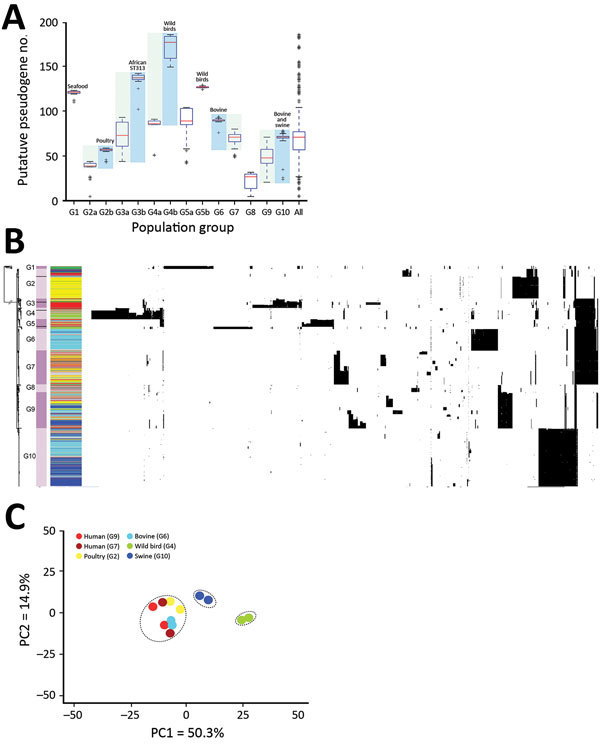
Pseudogene accumulation and metabolic acclimation of *Salmonella enterica* serotype Typhimurium. A) Abundance of putative pseudogenes in each individual population group or subgroup. Colors indicate each pair of recently diverged clades: light blue indicates source-associated clade; light green indicates diverse-source clade. B) Distribution of putative pseudogenes among *Salmonella* Typhimurium genomes by source. Cyan, bovine; yellow, poultry; light green, wild bird; blue, swine; dark green, miscellaneous food; red, human; gray, other sources. Purple bars delineate different population groups; black lines within these bars indicate subgroup divisions: G2a and G2b, G3a and G3b, G4a and G4b, and G5a and G5b. The presence of a pseudogene in an isolate is shown as a black spot in the corresponding location. Horizontally, these pseudogenes are hierarchically clustered on the basis of their distribution among analyzed isolates. C) Principal component analysis of metabolic profiles of selected isolates. Results from 2 replicate Phenotype Microarray (Biolog, https://biolog.com) analyses are shown for each isolate. PC, principal component.

We identified putative disruptive mutations (pseudogenes) in each *Salmonella* Typhimurium genome. Pseudogenes were most abundant in the sub-Saharan ST313 clade (G3b) and clades associated with wild birds (G4b and G5b) and seafood (G1). In multiple cases, we found significant differences (p<0.01) in pseudogene abundance between 2 recently diverged clades from a common ancestor; 1 comprised isolates from diverse sources and the other was associated predominantly with a particular source, such as wild birds (G4b), livestock (G2b, G6, and G10), and human ST313 cases in Africa (G3b) (Figure 2, panel A). We consistently observed elevated pseudogene accumulation in the source-associated and putatively host-adapted clades (Appendix 2 section 7). Most of these pseudogenes were clade-specific (Figure 2, panel B), suggesting they emerged and accumulated independently in different clades.

### Differential Metabolic Potentials Among Representative Isolates

We characterized metabolic profiles of 6 representative human and animal isolates from 6 US population groups (G2b, G4b, G6, G7, G9, and G10) using Biolog Phenotype Microarrays comprising substrates of carbon, nitrogen, sulfur, and phosphorus sources. We found evidence for differential metabolic activity between any 2 isolates for 189 of the 384 substrates tested (Appendix 1 Table 2). Principal component analysis on metabolic activities suggested that a wild bird isolate (STM223) from G4b and swine isolate from G10 (STM712) deviated from each other and the rest of the isolates because of deficiency in using multiple substrates (Figure 2, panel C). Compared with a human isolate from G9 (STM988), the wild bird isolate showed reduced metabolic activity for 182 substrates and the swine isolate for 132 substrates. Some of these deficiencies correlated with the putative pseudogenes and nonsynonymous SNPs (Appendix 2 section 8).

### Source Prediction Using WGS

To evaluate *Salmonella* Typhimurium source prediction using WGS, we updated the *Salmonella* Typhimurium genome collection (initially 1,267 genomes) by 1) adding 939 genomes that became available in GenomeTrakr during September 2015–January 2017; 2) sequencing another 11 isolates from 5 outbreaks with confirmed livestock origin in the United States during 2007–2013, which, together with 6 livestock isolates from 3 outbreaks in the original dataset, led to 8 zoonotic outbreaks for retrospective source attribution; and 3) excluding 744 redundant genomes to minimize biases resulting from repeated sampling of closely related strains. The genome updates resulted in a modified collection of 1,473 isolates for source prediction, all belonging to the previously defined 10 populations groups.

We observed phylogenetic clustering of isolates from the same zoonotic source (Figure 1, panel A). In general, 70.3% of isolates from bovine, poultry, swine, or wild bird (BPSW) sources shared the MRCA with an isolate from the same source, suggesting the possibility of source prediction by phylogenetic placement. By assigning a query outbreak isolate to the source of the livestock isolate that shared the MRCA with the query, we correctly predicted zoonotic sources for 6 of the 8 zoonotic outbreaks. Most of the reference isolates for source prediction were epidemiologically unrelated to the query isolates (i.e., separated by years [Table 1]). By contrast, only 36.9% of human isolates and 45.5% of food isolates (excluding G1) were from the same source as their nearest phylogenetic neighbor.

**Table 1:** Retrospective source attribution of zoonotic outbreaks of *Salmonella enterica* serotype Typhimurium, United States*

Isolate	Outbreak			Phylogenetic reference†			Population group	Phylogeny prediction	RF prediction
Year	Confirmed vehicle	Isolate	Year	Source
STM2207	2013	Ground beef		STM296	2006	Bovine	G9	+	+
STM2208	2013	Ground beef		STM296	2006	Bovine	G9	+	+
STM2209	2007	Pot pie turkey		STM093	2005	Poultry	G7	+	+
STM2210	2007	Pot pie turkey		STM093	2005	Poultry	G7	+	+
STM2211	2007	Pot pie turkey		STM093	2005	Poultry	G7	+	+
STM2212	2007	Pot pie turkey		STM093	2005	Poultry	G7	+	+
STM2213	2013	Live poultry		STM2114	2016	Bovine	G7	–	+
STM2214	2013	Live poultry		STM2114	2016	Bovine	G7	–	+
STM2215	2011	Ground beef		STM1563	2011	Bovine	G6	+	+
STM2216	2011	Ground beef		STM1563	2011	Bovine	G6	+	+
STM2217	2015	Pork		STM2116	2016	Swine	G2a	+	+
STM1016	2010	Cattle contact		STM328	2008	Bovine	G9	+	+
STM1075	2010	Cattle contact		STM978	2010	Bovine	G2a	+	+
STM995	2010	Cattle contact		STM978	2010	Bovine	G2a	+	–
STM996	2010	Cattle contact		STM978	2010	Bovine	G2a	+	–
STM1065	1998	Raw milk		STM034	2004	Bovine	G9	+	+
STM988	2009	Chicken		STM1975	2015	Bovine	G9	–	+

*RF, Random Forest; +, correct prediction; –, incorrect prediction. †The livestock genome that had the most recent common ancestor with an outbreak query genome.

In addition to overall phylogeny, we evaluated how the assortment of certain genetic features into particular *Salmonella* Typhimurium genomes could help predict zoonotic sources of *Salmonella* Typhimurium. Using a machine learning approach, we built a Random Forest (RF) classifier to interrogate a comprehensive collection of 3,137 genetic features among *Salmonella* Typhimurium genomes: core genome SNPs (n = 1,882), high-quality insertion/deletions (indels; n = 150), and source discriminatory accessory genes (n = 1,105). Trained by genomes of known BPSW origins, the classifier produced an out-of-bag accuracy rate (https://www.stat.berkeley.edu/~breiman/OOBestimation.pdf) of 82.9%. Seven of the 8 zoonotic outbreaks were attributed to the correct source by the RF method (Table 1).

**Figure 3:**
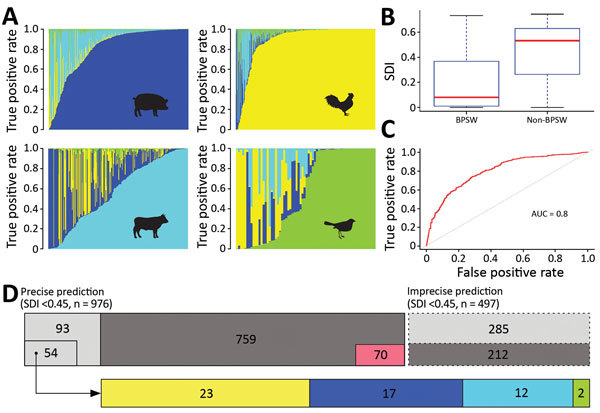
Source prediction by Random Forest classifier. A) Predicted source probabilities for zoonotic *Salmonella enterica* serotype Typhimurium isolates. Each vertical line in a panel is color coded by predicted source probabilities to proportion: cyan, bovine; yellow, poultry; blue, swine; light green, wild bird. B) Comparison of SDIs of predicted probabilities between BPSW and non-BPSW isolates. For each isolate, SDI was calculated among predicted probabilities of the 4 sources. Red horizontal lines indicate median SDI values; blue box tops and bottoms indicate interquartile ranges; whiskers indicate maximum and minimum SDI values. C) Receiver operating characteristics (ROC) curve of differentiating BPSW and non-BPSW isolates using SDI of predicted source probabilities. The AUC was 0.8, suggesting good binary classification. Red line indicates ROC curve; dotted line indicates diagonal line across the ROC space. D) Summary of source prediction results of 1,473 *Salmonella* Typhimurium isolates. Rectangles with solid and dashed lines represent precise (SDI <0.45) and imprecise (SDI >0.45) predictions, respectively. Dark gray rectangles, BPSW isolates; light gray rectangles, non-BPSW isolates. The number in each enclosed area is the number of isolates in the category. The sizes of enclosed and gray areas are in proportion to the numbers of isolates they represent. The 70 precisely but incorrectly predicted BPSW isolates are shown with outline. The 51 precisely predicted human isolates were attributed to zoonotic sources: cyan, bovine; yellow, poultry; blue, swine; light green, wild bird. The sizes of source colored rectangles are proportional to the numbers of isolates in the predicted source classes. AUC, area under the ROC curve; BPSW, bovine, poultry, swine, or wild bird; SDI, Simpson diversity index.

Among BPSW, the classifier performed best in predicting poultry and swine sources, followed by bovine and wild bird sources (Figure 3, panel A). This result was consistent with relative sampling intensities of these sources. Rarefaction analysis suggested that phylogenetic diversity was better sampled for poultry and swine than bovine and wild bird sources; wild bird *Salmonella* Typhimurium populations were least sampled (Appendix 2 section 9). For each isolate analyzed, the RF classifier generated a predicted probability for each source class. Because the classifier was not trained by isolates from non-BPSW sources, source prediction ambiguities were anticipated when it was used to predict non-BPSW isolates. Such ambiguities might present themselves as increased uncertainty in source prediction. We used the Simpson diversity indices (SDI) to measure the uncertainty of predicted probabilities between BPSW and non-BPSW isolates. The SDI of the non-BPSW group was significantly higher (p<0.01) than that of the BPSW group (Figure 3, panel B). We further showed that SDI of predicted probabilities made an effective binary classifier to distinguish BPSW from non-BPSW isolates in the current dataset through a receiver operating characteristic analysis, which yielded a sensitivity of 0.80 and a specificity of 0.63 adopting an arbitrary SDI cutoff of 0.45 (Figure 3, panel C).

Using this cutoff, we categorized source predictions into precise and imprecise groups (Figure 3, panel D). Precise predictions (SDI <0.45) were generated for 829 of 1,041 BPSW isolates, of which 759 (91.6%) were correct. A total of 147 non-BPSW isolates were precisely attributed to a BPSW source; among these, 51 were human isolates, which accounted for 31.9% of human isolates in the *Salmonella* Typhimurium collection.

Zoonotic source predictions by phylogenetic placement and RF analysis were generally consistent (Appendix 1 Table 6). Among the 829 isolates with precise predictions by the RF classifier, 705 (85.0%) isolates were correctly predicted by both methods. Detailed comparison of the 2 methods can be found in Appendix 2, section 10.

### Genetic Indicators of Source Association

**Figure 4:**
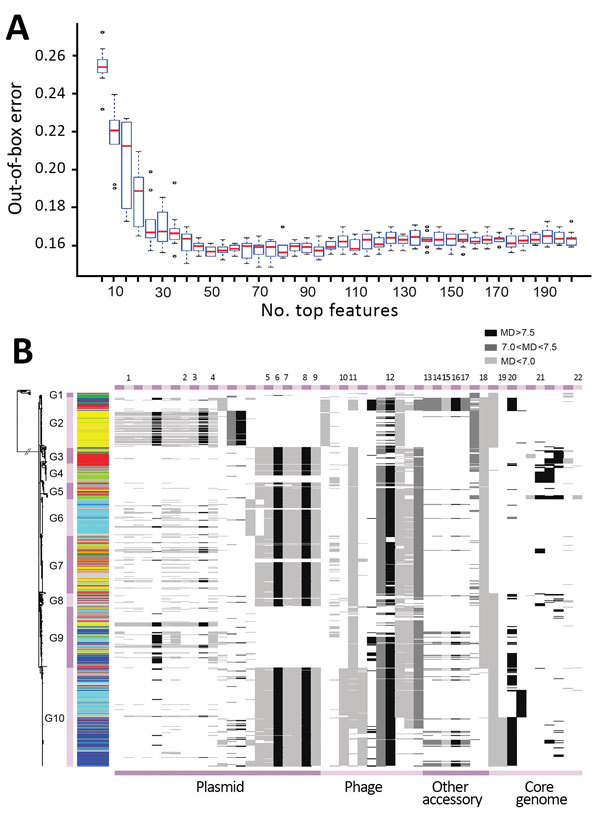
Key genetic features for zoonotic source prediction of *Salmonella enterica* serotype Typhimurium using Random Forest classifier. A) Change of out-of-bag prediction error rate as incremental inclusion of top ranking genetic features for source prediction. Red lines indicate median values; blue boxes indicate interquartile ranges. Upper and lower whiskers indicate maximum and minimum values. Circles indicate outliers. B) Distribution of top 50 source predicting features among *Salmonella* Typhimurium isolates on the basis of their location. Cyan, bovine; yellow, poultry; light green, wild bird; blue, swine; dark green, miscellaneous food; red, human; gray, other sources. The presence of a feature in an isolate is shown as a horizontal line in the corresponding location, with its grayscale representing the level of the MD of prediction accuracy through randomly permuting values of the feature. The higher the MD, the more important the feature is for source prediction. MD, mean decrease.

Using RF, we ranked all 3,137 genetic features by their importance for source prediction, which was measured by the mean decrease of prediction accuracy through randomly permuting feature values (Appendix 1 Table 7). To identify a subset of key features for source prediction, we incrementally incorporated features into the classifier based on their importance ranking and monitored the change of out-of-bag error rate. After an initial sharp drop, the error rate plateaued when ≈50 top-ranking features were included (Figure 4, panel A). Ten core genome mutations were among the top 50 features, 3 nonsynonymous SNPs and 7 indels (Figure 4, panel B). The 2 most important features for source prediction were nonsynonymous SNPs and related to cell surface components (Table 2). One of these was in the *fliC* gene, which is responsible for *Salmonella* flagellum formation and serotype determination. Forty of the other top 50 features were accessory genes found on plasmid- or phage-associated sequences (Figure 4, panel B). Several of these genes were involved in *Salmonella* interaction with host and environment, such as virulence genes *spvB* (*23*), *spvD* (*22*), and *pipB2* (*20*); virulence and putative host range factor *sspH2* (*19*); and multiple resistance genes to silver and copper (Table 2). Both elements are used as dietary supplements or antimicrobial drugs in livestock production (*24*,*25*). Another highly ranked accessory gene, *proQ*, was recently discovered to mediate global posttranscriptional regulation of gene expression (*21*). Twenty-four of the top 50 feature-related genes had been functionally tested for mediating intestinal colonization of livestock animals; 14 showed positive evidence for such a role (*9*). Although not ranked among the top 50 features, antimicrobial resistance genes exhibited enrichment and source-associated distribution patterns among livestock clades (Appendix 2 section 12).

**Table 2:** Selected key genetic features for zoonotic source prediction of *Salmonella enterica* serotype Typhimurium using a Random Forest classifier

Feature rank*	Affected gene	Feature type	Gene function (reference)
1	*fliC*	Single-nucleotide polymorphism	Motility, serotype diversity, intestinal colonization (*16*)
5	*traA*	Accessory gene	Pilin precursor. intestinal colonization (*16*)
6	*spvB*	Accessory gene	Virulence (*17*), intestinal colonization (*16*)
9	1930†	Accessory gene	Intestinal colonization (*16*)
11	1874†	Indel	Intestinal colonization (*16*)
13–15, 28	*cusCFBA*	Accessory gene	Putative copper efflux system (*18*)
16	*silP*	Accessory gene	Silver efflux pump (*17*)
21	*yafA*	Accessory gene	Intestinal colonization (*16*)
24	*sspH2*	Accessory gene	Virulence and potential host range factor (*19*)
27	0286A†	Accessory gene	Intestinal colonization (*16*)
31	*pipB2*	Accessory gene	Virulence (*20*)
32	*proQ*	Accessory gene	Global posttranscription regulation (*21*), intestinal colonization (*16*)
34	*spvD*	Accessory gene	Virulence (*22*)
37	*Trap*	Accessory gene	Intestinal colonization (*16*)
39	*yhfL*	Indel	Intestinal colonization (*16*)
41	0835†	Accessory gene	Intestinal colonization (*16*)
43	*traJ*	Accessory gene	Intestinal colonization (*16*)
45	*yceA*	Accessory gene	Intestinal colonization (*16*)
46	*exc*	Accessory gene	Intestinal colonization (*16*)

*Features are ranked by the mean decrease of prediction accuracy through randomly permuting values of the feature. The larger the mean decrease, the higher the rank. Only features that are located in genes that have reported involvement in intestinal colonization, virulence, and other functions related to livestock environment adaptation are listed. The full list of analyzed features, including the top 50 for zoonotic source prediction, is provided in Appendix 1 Table 6. Indel, insertion/deletion. †Locus identification of the reference genome SL1344.

## Discussion

Based on our determination of the large-scale phylogeny of *Salmonella* Typhimurium, including dense sampling of US livestock isolates, we speculate emerging host adaptation associated with livestock production. Evidence to support this hypothesis includes increasing pseudogene accumulation as livestock clades diverged and metabolic deviation of a representative swine isolate. Both phenomena have been reported as possible signs of *Salmonella* host adaptation (*26*–*29*). These adaptation signals were detectable but moderate compared with the wild bird and ST313 clades, indicating emerging adaptation. Major clades of livestock isolates (G2b, G6, and G10) occupied terminal branches of the phylogeny and shared recent common ancestors with diverse-source clades, suggesting their recent emergence through clonal expansion. An exhaustive screening for temporal signals throughout the phylogeny enabled MRCA dating of 2 livestock clades in G2b and G10 around the 1990s, supporting their recent origin. The G10 clade contained the swine isolate displaying putative metabolic acclimation, as well as definitive type 104 isolates (Figure 1, panel B), whose global circulation started around the 1990s (*5*), consistent with our MRCA dating.

Major livestock clades all included animal isolates across the United States and spanned >10 years. Phylogenetic clustering of isolates spanning a wide geotemporal range also has been noticed in poultry-related *Salmonella* Enteritidis in the United States (*30*). The dissemination of closely related *Salmonella* strains from specific sources in the poultry sector could explain these observations.

Host prediction of *Salmonella* and *Escherichia coli* genomes through a machine-learning approach has been recently reported (*31*,*32*). A machine-learning classifier is inherently constrained by the representativeness of its training classes. However, for application in a realistic source attribution scenario, a classifier would be applied prospectively to isolates from sources a priori unknown to the classifier. Unlike those used in previous studies, our RF classifier was tested by isolates from various nontraining sources and capable of flagging them as imprecise predictions. This distinction might be useful for analyzing foodborne pathogens of a wide source range.

A previous support vector machine classifier had >90% accuracy in predicting the human host of *Salmonella* Typhimurium isolates using WGS data (*32*). Although human is not a source category in foodborne pathogen source attribution studies, which aim to attribute known human isolates to food and other sources, we performed a similar host specificity prediction using our RF classifier and *Salmonella* Typhimurium dataset. Only 36.9% of human isolates in our study were predicted to originate from a human host (Appendix 2 section 11). We found that the higher accuracy of human host prediction by the support vector machine classifier (*32*) was due mainly to an exceedingly clonal structure of its training human isolates, of which 85% shared the MRCA with another human isolate, compared with only 36.9% in our training set (Appendix 2 section 13). The percentage for the training set was consistent with our sampling of diverse human isolates in the United States based on surveillance data, including molecular subtypes. To avoid inflating source prediction accuracy by overrepresenting closely related genomes in the training set, we reduced training data redundancy by excluding 744 genomes from all training classes based on their pairwise phylogenetic distance and strain metadata (Appendix 2 section 6). Our classifier could not distinguish US human isolates from isolates of other sources by genomewide analysis of genetic features, arguing against distinct human host signals in *Salmonella* Typhimurium genomes or suggesting that the human isolates represent a mixture of strains immediately derived from multiple other sources. Higher occurrence of certain *Salmonella* Typhimurium subtypes in human cases, therefore, more likely results from their prevalent circulation in foods and the environment, as observed in G7, than from elevated infectivity or virulence.

The known zoonotic hosts and reservoirs of *Salmonella* Typhimurium appeared to be highly attributable. When the classifier was precise about a BPSW origin, 91.6% of isolates were correctly predicted. A narrow coverage of livestock isolate diversity in the United States could skew prediction accuracy. This scenario was countered by the inclusion of isolates from major US *Salmonella* Typhimurium outbreaks of livestock origins over 15 years, most of which our classifier correctly predicted. Furthermore, livestock populations of *Salmonella* Typhimurium appeared to be more clonal than human isolates (Figure 1, panel A), possibly in association with industrialized livestock production.

Our classifier performed genomewide, high-resolution interrogations of genetic features, including not only accessory genes as previously reported (*31*,*32*) but also core genome mutations, such as SNPs and indels. This approach led to the discovery of a point mutation in *fliC* that outperformed all the other features in source prediction. *fliC* encodes the filament portion of the bacterial flagella, flagellin. Flagellin shows substantial antigenic diversity across *Salmonella* that has been exploited for *Salmonella* serotyping. It remains unclear whether the 2 different FliC proteins we discovered have different biologic properties that might correlate to zoonotic source. Our demonstration that a few key genetic features were sufficient for robust source prediction paves the way for developing efficient source attribution models scalable to large and expanding volumes of WGS data, which in turn is likely to improve the training and performance of the RF classifier.

Our classifier and pilot source attribution study based on the current training set is limited to predicting major livestock sources of *Salmonella* Typhimurium. Fewer than one third (31.9%) of human isolates were precisely attributed to a BPSW source, indicating additional sources of human salmonellosis underrepresented by the current training set. Continuing accumulation of *Salmonella* Typhimurium genomes from environmental sources might be expected to lead to the identification of additional source classes to enable source attribution of more human clinical isolates using the machine-learning approach and could potentially help generate source hypotheses for outbreak investigation. Regular update of the training set is required for incremental improvement of the classifier, particularly for tracking new and emerging strains.

Another limitation is related to the ability of certain *Salmonella* Typhimurium isolates to circulate among multiple sources, which challenges precise source prediction. For example, source prediction of BPSW isolates in the diverse-source G7 lineage was lower at 64.2% than the out-of-bag prediction accuracy at 82.9% of all BPSW isolates. Nevertheless, G7 isolates from the 2009 turkey pot pie–associated outbreak and the 2013 live poultry–associated outbreak were attributed to poultry. These successful attributions were most likely due to the inclusion of other G7 poultry isolates in the training set that informed the classifier. Growing availability of training genomes in generalist lineages may reveal fine-scale clustering of *Salmonella* Typhimurium isolates by source to help source prediction.

Finally, transmission of *Salmonella* from a zoonotic source to a nonanimal food vehicle is possible. For example, domesticated and wild animals can cause *Salmonella* contamination of irrigation water, which may subsequently contaminate fresh produce (*33*). Two G7 outbreaks with precise source predictions were linked to fresh produce, the 2010–2011 multistate alfalfa sprout–associated outbreak and the 2012 multistate cantaloupe-associated outbreak. In both cases, the outbreak isolates were attributed to poultry. Although the findings cannot be confirmed, our study provides a potential new tool to help identify root sources of foodborne *Salmonella* Typhimurium outbreaks.

Appendix 1 Additional tables for zoonotic source attribution of *Salmonella enterica* serotype Typhimurium using genomic surveillance data, United States.

Appendix 2 Additional methods and results for zoonotic source attribution of *Salmonella enterica* serotype Typhimurium using genomic surveillance data, United States.
