# Supplementary material for: Zoonotic Source Attribution of Salmonella enterica Serotype Typhimurium Using Genomic Surveillance Data, United States
Source: Emerg Infect Dis. 2019 Jan;25(1):82–91. doi: 10.3201/eid2501.180835 (PMC6302586; doi:10.3201/eid2501.180835)
Supplement: Appendix 2 — Additional methods and results for zoonotic source attribution of Salmonella enterica serotype Typhimurium using genomic surveillance data, United States. [file 18-0835-Techapp-s2.pdf]

Article DOI: https://doi.org/10.3201/eid2501.180835

Zoonotic Source Attribution of *Salmonella enterica* Serotype Typhimurium Using Genomic Surveillance Data, United States

Appendix 2

1. Sequencing and Selection of *Salmonella enterica* serotype Typhimurium Genomes

A total of 127 human clinical isolates were grown in tryptic soy broth (TSB) overnight. Genomic DNA was extracted by using the GenElute Genomic DNA isolation kit (Sigma-Aldrich, St. Louis, MO, USA). Sequencing libraries were prepared using Illumina TruSeq DNA library kit (San Diego, CA, USA) and sequenced on an Illumina HiSeq instrument (San Diego, CA, USA) according to manufacturer's instruction. Trimmomatic v0.32 (*1*) was used to remove low-quality reads. The leading 3 and the trailing 3 nucleotides were removed from the reads, and a 4-nucleotide sliding window was used to remove nucleotides from the 3′ ends when the average Phred score dropped below 20. Then the raw reads were assembled into draft genomes using SPAdes v3.7.0 by default settings (*2*). The quality of publicly available *S. enterica* Typhimurium draft genomes used in this study was assessed by QUAST v4.5 (*3*). Assemblies with an N50 value <100,000 were excluded from further analysis.

2. Phylogenetic Analysis

Genome regions related to repetitive sequences, phages, and recombination were removed from the reference genome prior to phylogeny construction. Repetitive and phage sequences were detected in the reference SL1344 genome (NCBI accession: NC_016810) by MUMmer v3.23 (*4*) and Phast (*5*), respectively. Recombinant sequences were identified by taking the union of the inferences by Gubbins v2.2.0 (*6*) and ClonalFrameML v1.25 (*7*). Genomes in the G1 group were excluded from recombination analysis because their phylogenetic distance with the rest of *S. enterica* Typhimurium genomes was too distant for the recombination

detection tools to properly detect recombination. Draft genomes were assembled from raw sequencing reads using SPAdes (*2*). A maximum-likelihood phylogenetic tree was constructed by FastTree2 v2.1.7 (*8*) based on core genome alignment of draft and finished *S. enterica* Typhimurium genomes through Parsnp v1.2 (*9*). The modified SL1344 genome free of inferred repetitive, phage, and recombinant sequences was used as a reference for the alignment. Major population groups were identified using BAPS v6.0 (*10*). Two levels with a maximum 50 populations were set as the initial condition for BAPS to infer population structure of *S. enterica* Typhimurium genomes using a maximum-likelihood approach.

3. Temporal Signal Identification and MRCA Dating

To preliminarily explore local temporal signals of SNP accumulation throughout the global phylogeny of *S. enterica* Typhimurium, every subtree (i.e. internal node) of the phylogeny was screened by calculating the Spearman's correlation coefficient and the Pearson's correlation coefficient between isolation years of the corresponding isolates and tip-to-root (i.e., the internal node) distances of these isolates. We used both Spearman's and Pearson's correlation coefficients as preliminary screening metrics to maximize the discovery of candidate subtrees displaying temporal signals of SNP accumulation. Subtrees whose squared coefficient (R2) of either the Spearman's or the Pearson's correlation exceeded 0.4 were selected for further temporal analysis. Pearson's correlation was used as a secondary screening metric; if an internal node had a R2 over 0.4 for Spearman's, Pearson's correlation was not calculated. Raw reads of genomes in each candidate subtree were first aligned to their nearest finished genome on the global phylogeny using BWA v0.7.12 (*11*). The use of a closely related reference genome when available maximized the detection of SNPs present among the members of a subtree, but absent from a more distant reference genome. High quality SNPs were called using FreeBayes v1.1.0-44 (*12*). These SNPs were defined to meet 3 criteria: 1) a minimum of 5 reads were mapped to the SNP locus, 2) at least 75% of mapped reads supported the SNP calling, and 3) a minimum mapping quality score generated by BWA at the SNP locus was 20. Concatenated high quality SNPs were used to construct a maximum-likelihood tree by Phyml v20120412 (*13*). Then, TempEst v1.5 (*14*) was used to repeat the calculation of the linear coefficient between isolation years and branch lengths of candidate subtrees to confirm their temporal signals. Any subtree with R2 >0.4 was considered to display a temporal signal and then subjected to model-based

population dynamics analysis using BEAST v1.8.2 (*15*) with at least 200,000,000 states and sampling of every 5,000. The maximum clade credibility tree was generated by TreeAnnotator v1.8.2 (http://beast.bio.ed.ac.uk/TreeAnnotator) to estimate the ages of most recent common ancestors. Model performance was assessed through Tracer v1.6 (http://beast.bio.ed.ac.uk/Tracer). Four different combinations of tree and clock models were tested for each clade and compared through Bayes factors (BF) (*16*): 1) Gaussian Markov Random Fields (GMRF) as tree model and relaxed log-normal molecular clock as clock model, 2) GMRF as tree model and strict clock rate as clock model, 3) constant population model as tree model and relaxed log-normal molecular clock as clock model, and 4) constant population model as tree model and strict clock rate as clock model. The combination with the highest Bayes factor (BF) was selected for analysis. As shown in Appendix 1 Table 8 (https://wwwnc.cdc.gov/EID/article/25/1/18-0835-App1.xlsx), the combination of Gaussian Markov Random Fields (GMRF) and relaxed log-normal molecular clock was favored for a poultry clade in G2 and a swine clade in G10 by yielding the highest BF among model combinations. For another swine clade in G9, the combination of constant effective population size and relaxed log-normal molecular clock was favored. For all 3 clades, a relaxed log-normal molecular clock was favored over a strict clock rate (log10BF between 17 and 50), suggesting that mutation rates vary among branches. This was consistent with our observation that temporal signal of SNP accumulation was only evident locally in certain clades instead of globally across the entire tree. Estimated substitution rate and most recent common ancestor age for each clade were summarized in Appendix 1 Table 5.

4. Identification of putative pseudogenes

Putative core genome pseudogenes were defined by having any of the following mutations: 1) nonsynonymous SNPs (NS-SNPs) in the start or stop codon; 2) frameshift mutations caused by indels; 3) truncations that spanned at least 20% of a coding region; 4) NS-SNPs or non-frameshift indels that were potentially deleterious (explained below); and 5) premature stop codons. Specifically, indels were identified from raw reads by Scalpel v0.5.3 (*17*) using SL1344 as the reference genome and confirmed by manual inspection. A preliminary indel was first called if 1) a minimum of 5 reads were mapped the locus of the indel, and 2) at least 50% of mapped reads supported the indel calling. High fidelity indels were confirmed through

comparison with corresponding sequences in the reference genome. The reference genome sequence between 100 bp upstream and 100 bp downstream of a preliminary indel was extracted and compared with the draft genome of the *S. enterica* Typhimurium isolate in which the indel was identified. The comparison was performed by BLAST, and the result was manually inspected. Deleterious NS-SNPs were identified by Provean v1.1.5 (*18*). Any NS-SNP with a Provean score lower than a default threshold of –2.5 was considered to be deleterious. To identify gene truncations, the predominant allele of each core gene identified by Roary v3.8.2 (*19*) from de novo assemblies was determined. Any allele with at least 20% of its coding region deleted in comparison with the predominant allele was considered to be truncated. NS-SNPs in start and stop codons and premature stop codons were identified by locating the mutations in the annotated SL1344 reference genome.

When SL1344 (or any genome) is used as the reference to identify mutations in other genomes that may cause putative pseudogenes, it is assumed that alleles in the reference genome represent the wild type, non-mutant forms of the genes. This assumption may not apply to already mutated genes specific to the reference genome. Such reference-specific mutations need to be identified to correct the bias in pseudogene identification caused by the selection of reference genome. NS-SNPs that were present in 1%–99% of the 1,267 *S. enterica* Typhimurium genomes and all of the identified indels were examined for their distribution among the genomes. As shown in Appendix 2 Figure 1, a total of 138 SNPs and 21 indels (highlighted by red dashed box) were found in the majority of population groups, including the distantly related G1 but absent from G5a, in which the reference SL1344 genome was located, and/or a few other population groups that shared a recent common ancestor with G5a. The most parsimonious explanation for this distribution pattern is that these mutations are specific to G5a and its closely related groups, as compared with the less parsimonious hypothesis of multiple independent occurrences of the same mutations in different lineages. Therefore, for pseudogene identification, the SL1344 reference alleles that contained such mutations were replaced by the likely non-mutant alleles inferred from genomes free of these mutations. The final set of putative pseudogenes identified in this study is summarized in Appendix 1 Table 10.

5. Source Classification

Seven source classes were defined for source attribution analysis: 1) human, including human clinical isolates; 2) bovine, including isolates from cattle, beef, and raw milk; 3) poultry, including isolates from chicken, turkey, duck, and eggs; 4) wild birds, including isolates from wild bird species, such as sparrow and gull; 5) swine, including isolates from pigs and pork; 6) miscellaneous food, including seafood such as fish and shrimp, plant-based food such as grains and produce, and other ready-to-eat and/or processed food, such as peanut butter and cheese; and 7) others, including any isolates not belonging to the aforementioned classes. Bovine, poultry, and swine sources were categorized as livestock. Outbreak isolates with confirmed food or livestock origins were assigned to such sources.

6. Random Forest-based Source Attribution

The training set for the RF classifier contained a total of 1,041 genomes from BPSW sources (nbovine = 195, npoultry = 440, nswine = 338 and nwild birds = 68). A total of 2,217 *S. enterica* Typhimurium genomes were collected or sequenced in this study, including the original set of 1,267 genomes by September 2015, an addition of 939 genomes that became publicly available thereafter, and 11 outbreak genomes that were sequenced for retrospective investigation. To alleviate sampling biases due to repetitive inclusion of closely-related isolates, we identified isolates that were separated by a maximum of 10 core genome SNPs and isolated from the same source and in the same geographic location (a US state or a non-US country) and in the same calendar year. One representative was randomly selected from each cluster of closely-related isolates and kept in the study. The rest of the cluster were considered to be redundant and discarded. A total of 744 redundant genomes were removed, leading to a final set of 1,473 genomes for source attribution analysis, including the 1,041 BPSW isolates for classifier training.

To select genetic features for the RF classifier, SNPs, indels and accessory genes were identified from the updated collection of 1,473 non-redundant genomes. SNP, and indel identifications were performed as previously described. Accessory genes were defined as genes present in <99% of the 1,473 genomes. Draft genomes annotated by Prokka v1.12 (*20*) were analyzed by Roary (*19*) to identify accessory genes. A total of 34,892 SNPs, 213 indels, and

29,813 accessory genes were identified. Any SNP and indel that was unique to the non-US G1 group, located in intergenic regions, and present in <1% or >99% of genomes was excluded, leading to the final sets of 1,882 SNPs and 150 indels to be included as genetic features for the RF classifier. Because the sheer number of accessory genes (n = 29,813) was prohibitively large for the RF classifier, accessory genes were further filtered by their source discriminatory power among analyzed *S. enterica* Typhimurium isolates. Specifically, accessory genes unique to the non-US G1 group were excluded first. Then, we defined source prevalence of an accessory gene as the percentage of isolates from a particular source that had the gene. For any accessory gene, if its source prevalence differed by <25% between its most and least prevalent BPSW sources, the gene was considered to be insufficiently discriminatory among sources and removed. After removing these genes, a final set of 1,105 accessory genes were included as genetic features for the RF classifier.

The RF classifier was built using the randomForest package (v4.6-12) of R. The "ntree" argument was set to be 1,000, and default settings were used for other parameters. To estimate prediction errors and infer feature importance, the RF algorithm used out of the bag (OOB) samples created by bootstrap aggregating or bagging of training data (*21*).

7. Elevated Accumulation of Putative Pseudogenes Accompanied the Emergence of Putatively Host-Adapted Clades

Putative pseudogene formation was evident among sub-Saharan ST313 isolates and wild bird isolates. Evidence of host adaptation has been reported for isolates from these sources (*22*,*23*). We examined pseudogene accumulation during the evolutionary emergence of the ST313 clade (G3b) and a wild bird clade (G4b). Putative pseudogenes were identified as described in the Methods section of the main text to include 5 categories of mutations: 1) nonsynonymous SNPs (NS-SNPs) in the start or stop codon; 2) frameshift mutations caused by indels; 3) truncations that spanned at least 20% of a coding region; 4) NS-SNPs or non-frameshift indels that were potentially deleterious; and 5) premature stop codons. The abundance of putative pseudogenes was surveyed in individual clades that shared increasingly more recent common ancestors with the ST313 clade (Appendix 2 Figure 2, panel A) or the wild bird clade (Appendix 2 Figure 2, panel B). The 2 phylogenies shown in Appendix 2 Figure 2 were subtrees

from the entire *S. enterica* Typhimurium tree (Figure 1, panel A) that was mid-point rooted. In both instances, the divergence and evolution of putatively host-adapted lineages appeared to coincide with elevated accumulation of putative pseudogenes. As shown in Appendix 2 Figure 2, more recently diverged clades were associated with higher abundances of putative pseudogenes.

8. Inference of Genotypic Causes of Metabolic Differences

Overall metabolic potentials of 6 representative isolates from 6 major population groups (G2b, G4b, G6, G7, G9, and G10b; Appendix 1 Table 2) were evaluated using Biolog Phenotype Microarrays (PM) (Hayward, CA, USA) according to manufacturer's instruction. Phenotype MicroArray MicroPlates 1-4 (PM1-4) were used for the metabolic profiling. These plates include substrates of carbon (PM1 and PM2), nitrogen (PM3), and sulfur and phosphorus (PM4) sources. The metabolic profiling was performed at 37°C that was similar to body temperatures of humans and other warm-blooded animal hosts of *Salmonella*. Colorimetric measurement of each well was taken every 15 m for 48 h. Data analysis was performed using OmniLog PM System under the default setting. Principal component analysis (PCA) was conducted using the PM results. Among representative isolates from source-associated clades (STM096, STM223, STM481, and STM712), reduced or loss of ability to utilize certain substrates compared with the reference isolate (STM988/2009K-1063) was most evident for a swine isolate (STM712) and a wild bird isolate (STM223) according to the PM analysis (Figure 2, panel C; Appendix 1 Table 2). Their corresponding clades (G10 and G4b) exhibited elevated levels of putative pseudogene accumulation compared with diverse-source clades (Figure 2, panel A). We sought to identify potential genotypic causes for the different metabolic phenotypes. For a substrate that was differentially utilized between the swine/wild bird isolate and the reference isolate, genes involved in corresponding metabolic pathway were identified using KOBAS v3.0 (*24*) and KEGG (*25*). Putative pseudogenes and nonsynonymous SNPs that could potentially disrupt a metabolic pathway were searched in these genes. One example is shown in Appendix 2 Figure 3. It is not clear if the disrupted sulfate reduction pathway plays any role in host adaptation of *S. enterica* Typhimurium. Inferences of genotypic causes of metabolic deviation were summarized in Appendix 1 Table 9.

9. Rarefaction Analysis of Relative Sampling Intensity by Source

A rarefaction analysis was performed to assess relative sampling intensities by sources. Sampling richness was evaluated by measuring the quantity of phylogenetic clusters for each source. A phylogenetic cluster was defined as a monophyletic group of closely related isolates. Individual clusters were delineated by imposing a heuristically determined maximum pairwise SNP distance among isolates within a cluster. This heuristic value determined the sizes as well as the total number of phylogenetic clusters to be identified from the *S. enterica* Typhimurium phylogeny; the lower the value, the more clusters were found. To select a proper value that reflects phylogenetic distance among isolates from source-associated clades, we examined a total of 963 internal nodes from which a minimum of 5 isolates (external nodes) had descended and identified a total of 49 non-overlapping clades associated with bovine, poultry, swine, or wild bird (BPSW). Each of the 49 source-associated clades had at least 75% isolates from the same BPSW source. The maximum pairwise SNP distance in each of these clades was calculated. The mean of these distances was 150 SNPs, which led to the delineation of 205 phylogenetic clusters from the phylogeny of 2,217 genomes. The rarefaction analysis was performed on this dataset using the vegan package v2.4-4 (*26*) in R (*27*). The number of phylogenetic clusters represented by a certain number of isolates was examined for each source (Appendix 2 Figure 4, panel A). The steeper slopes of human and miscellaneous food curves suggested greater diversities were yet to be sampled from human and food isolates than BPSW isolates. The observation of more diverse clusters among human and food isolates also suggested diverse sources of *S. enterica* Typhimurium human infections and food contaminations in addition to BPSW. Less diversity is expected to be uncovered by continuing sampling of BPSW isolates than human and food isolates. Similar trends of relative sampling intensities were observed by adjusting the SNP distance threshold for phylogenetic cluster definition from 150 to 50, 100 and 200 (Appendix 2 Figure 4, panels B–D).

10. Comparison between *S. enterica* Typhimurium Zoonotic Source Predictions Using Phylogenetic Placement and RF Classifier

Among the 829 BPSW isolates that were precisely predicted (Simpson index of predicted source probabilities <0.45), 52 (6.3%) were incorrectly predicted by both RF and phylogenetic

placement (PP), 54 (6.5%) were incorrectly predicted by PP alone, and 18 (2.2%) were incorrectly predicted by RF alone. Source prediction by PP was performed by assigning a query to the source of the livestock isolate that shared the MRCA with the query. A maximum-likelihood phylogeny of *S. enterica* Typhimurium isolates used for source prediction analysis can be viewed at https://itol.embl.de/tree/19813720218255981525121446. An alternative method for source prediction by PP is to identify the predominant source in the clade in which a query isolate is placed and assign the source to the query. This method is algorithmically more complex by requiring arbitrary definitions of clades and source predomination threshold. It is also presumably more sensitive to sampling biases that lead to over- or under-representation of isolates from certain sources and clades, which are commonly found in public depositaries of pathogen genomes.

Incorrect Source Predictions by Both RF and PP (n = 52)

On the phylogenetic tree, nearly all the isolates whose sources were incorrectly predicted by both methods were found in clades that were dominated by isolates from the predicted source. Such cases may be explained by spillover transmissions between different sources. Putative spillover events may have occurred between bovine and swine (n = 20, STM452, STM2167, STM325, STM184, STM027, STM696, STM1173, STM443, STM1661, STM512, STM455, STM471, STM335, STM1359, STM453, STM1620, STM755, STM2004, STM1665, STM1817), poultry and wild bird (n = 10, STM877, STM265, STM224, STM228, STM263, STM257, STM261, STM256, STM258, STM262), swine and poultry (n = 7, STM177, STM1510, STM067, STM1227, STM170, STM1175, STM815), bovine and poultry (n = 1, STM968), bovine and wild birds (n = 1, STM457), and swine and wild birds (n = 2, STM649, STM259). Appendix 2 Figure 5, panel B, shows an example of incorrect prediction caused by a putative spillover infection between wild bird and swine. The isolate involved (STM649) was incorrectly predicted by both methods as wild bird.

A total of 11 genomes (STM433, STM1911, STM266, STM2077, STM294, STM298, STM287, STM260, STM1246, STM166, STM791) for which the source was incorrectly predicted by both methods belonged to clades of diverse sources. These incorrect predictions suggested the challenge for WGS-based source prediction due to likely generalist strains that can be associated with different sources and hosts.

Correct Source Prediction by RF but Incorrect Prediction by PP (n = 54)

Of the 54 isolates whose BPSW sources were correctly predicted by RF but incorrectly predicted by PP, 47 might be attributed to interference caused by putative spillover events that confounded the PP method (STM715, STM704, STM1473, STM754, STM721, STM329, STM327, STM710, STM708, STM1168, STM861, STM727, STM1874, STM1446, STM1952, STM777, STM1986, STM2190, STM2002, STM215, STM204, STM463, STM1162, STM447, STM1416, STM342, STM062, STM149, STM1811, STM1774, STM1262, STM1827, STM1803, STM2116, STM1452, STM1542, STM2064, STM1730, STM1899, STM2147, STM2154, STM1954, STM2039, STM2163, STM1534, STM1775, and STM803). For example, as shown in Appendix 2 Figure 5, panel B, an isolate in a wild bird clade might be transmitted to a swine host (STM649). By PP, the swine label of the transmitted isolate would lead to an incorrect swine prediction of its neighboring isolate (STM204) in the original wild bird clade. RF prediction by contrast was not affected because it was based on the strong wild bird signal of STM204 and the rest of the wild bird isolates in the clade.

Another 6 isolates were correctly predicted by RF despite falling into a mixed-source clade (STM477, STM398, STM179, STM155, STM835 and STM284). One example, STM477, is shown in Appendix 2 Figure 5, panel A. It was correctly predicted to be bovine by RF even though it was located in a diverse-source clade including poultry, wild bird, and swine. One additional outlier isolate (STM1945) was distantly related to any BPSW isolate but still correctly predicted by RF. These predictions suggested that the RF classifier was able to function in some cases where lack of phylogenetic references might present a challenge to source prediction by PP. These RF predictions were based on compositions of genetic features similar to those of the training genomes from the same source. The fact that the majority of top 50 predictor features used by RF were located on mobile genetic elements (Figure 4, panel B) indicates that genes that can be horizontally transferred may at least partially contribute to RF’s superior performance in such cases.

Correct Source Prediction by PP but Incorrect by RF (n = 18)

Six isolates in this category were found in clades where isolates from both bovine and poultry were present (STM174, STM299, STM295, STM326, STM1232, STM1806). Nine isolates were located in clades that were dominated by isolates from a different source (STM324, STM301, STM456, STM1639, STM995, STM996, STM978, STM1035, and STM267). Such

cases could be explained by putative spillover events that involved >1 closely related isolates, which served as the phylogenetic reference for each other to allow source prediction by PP as shown by STM324 and STM517 in Appendix 2 Figure 5, panel C. Three bovine isolates (STM302, STM459, STM278) were incorrectly predicted by RF as poultry or swine in spite of falling into mostly bovine clades. Interference from nearby poultry or swine isolates carrying similar genetic features as the query bovine isolates in their respective clade may cause the incorrect RF predictions.

11. Evaluation of Human Host Prediction Using the Random Forest (RF) Classifier

We performed an evaluation on predicting human host of *S. enterica* Typhimurium isolates using the Random Forest (RF) classifier similar to a previous study reported by Lupolova et al. that was based on a Support Vector Machine (SVM) classifier (*28*). For this analysis, human clinical isolates in the dataset (n = 160) were used as a separate training class. Miscellaneous food isolates, which displayed similar phylogenetic diversity as human isolates, were also included in the new classier as a training class for comparison. Human, miscellaneous food along with the 4 original classes of BPSW made the 6 training classes for the new classifier. After excluding redundant genomes that were separated by <10 SNPs and sampled from the same source and location in the same calendar year, a total of 1,473 genomes were included in the training data set. The same sets of core genome SNPs (n = 1,882) and indels (n = 150) for the BPSW classifier were used for the new classifier. Accessory genes were identified from the training *S. enterica* Typhimurium genomes using Roary (*19*) and defined as genes present in <99% of the genomes. For any accessory gene, if its source prevalence (defined as the percentage of isolates from a particular source that had the gene) differed by <25% between its most and least prevalent sources including human, food and BPSW, the gene was considered to be an insufficiently source discriminatory feature and removed from the analysis. After removing these genes, a final set of 1,282 accessory genes were obtained, a moderate increase from the 1,105 used by the BPSW classifier due to the inclusion of human and food isolates in the training data set. The RF classifier was built using the randomForest package (v4.6-12) (*28*) of R (*27*). The "ntree" argument was set to be 1,000 and default settings were used for other parameters. To estimate prediction errors, the RF algorithm used out of the bag (OOB) samples created by bootstrap aggregating or bagging of training data (*21*).

Preliminary evaluation of prediction accuracy for human host and miscellaneous food was 36.9% and 47.6% (excluding G1) respectively, compared with that between 50% and 90% for BPSW sources. The definition of the miscellaneous food source in this study included any food items other than retail meats. As the result, the food class defined here did not represent a singular and coherent reservoir of *S. enterica* Typhimurium. It was instead an aggregation of many food vehicles, of which any particular type did not have enough isolates to qualify as a single source class. Therefore, the low source prediction accuracy for food was likely the result of the inclusive classification of food. Human infections of *S. enterica* Typhimurium are mostly foodborne and *S. enterica* Typhimurium isolates should consequently reflect the diversity of miscellaneous food isolates, assuming *S. enterica* Typhimurium isolates circulating in foods are commonly virulent to humans. Human isolates in this study were indeed diverse and present in every population group along with miscellaneous food isolates, which explained the similarly low prediction accuracy for the human source.

12. Distribution of Acquired Antimicrobial Resistance Genes Among Zoonotic Sources of *S. enterica* Typhimurium

Antimicrobial resistance genes (ARGs) were identified from each of the 1,267 *S. enterica* Typhimurium genomes using the ResFinder database v3.0 (*29*). Each allele in the database was aligned to an *S. enterica* Typhimurium genome using BLAST. The presence of an ARG allele in the query genome was determined using default ResFinder setting. If an ARG type includes multiple alleles that showed at least 90% sequence similarity with each other, the alleles were clustered and 1 representative allele was used for alignment with the query genome. The distribution of detected ARGs among *S. enterica* Typhimurium genomes was shown in Appendix 2 Figure 6. Hierarchical clustering of detected ARGs was performed using the hclust package (*30*) of R (*27*). Distribution and clustering patterns of ARGs appeared to vary among zoonotic sources of *S. enterica* Typhimuiurm. Notably, ARGs were much less abundant in the wild bird clade compared with livestock clades. Antibiotic use in livestock may be related to the higher occurrence of ARGs in *S. enterica* Typhimurium isolates from livestock sources.

13. Different Levels of Human Isolate Clonality Between the Current and a Previous Machine Learning Classifier for *S. enterica* Typhimurium Source Prediction

While the RF classifier developed in this study and a Support Vector Machine (SVM) classifier reported in a previous study (*28*) both performed well in predicting livestock hosts or sources of *S. enterica* Typhimurium, their performances in predicting the human host of *S. enterica* Typhimurium genomes differed. Our RF classifier produced a host prediction accuracy of 36.9% for human isolates in our dataset compared with that of >90.0% by the SVM classifier using a different dataset. To investigate the cause of this difference, we compared the levels of human isolate clonality between the 2 studies. We constructed a maximum-likelihood tree based on core genome alignment of all the *S. enterica* Typhimurium genomes included in each study using Parsnp (*9*). As shown in Appendix 2 Figure 7, a total of 318 human isolates were included in the training set of the SVM classifier, most of which were clustered in 2 major clades. By contrast, a total of 160 human isolates used in the current study were scattered throughout the tree, with no visible clustering of many isolates except the Sub-Saharan Africa ST313 clade.

To exclude the possibility that the low accuracy in human host prediction was caused by the choice of RF as the machine learning classifier in the current study, we repeated the human host prediction analysis using SVM. We built a SVM classifier based on pan-genome gene content similar to the previously described approach (*28*) using the training set of the current study. Human and BPSW sources were included in the SVM classifier. The host prediction accuracy for human isolate was even lower at 13.5% by SVM compared with that of 36.9% by RF (or 35.6% if miscellaneous food isolates were excluded from the training set) (Appendix 1 Table 11). Similarly, SVM's performance in predicting BPSW source was lower than that of RF (Appendix 1 Table 11). In contrast with the systemic difference in performance between the 2 classifiers when applied to the current dataset, the relative difference between BPSW source prediction and human host prediction within a classifier was similar, with SVM being 46.6% and RF being 43.0% (Appendix 1 Table 11). These observations suggest that it was the dataset instead of the prediction algorithm that caused the difference in human host prediction between the current and the previous studies. Over-representation of clonally closely related isolates from a particular source in the training set can inflate the accuracy of machine learning-based

attribution to the source. As practiced in the current study, selection of phylogenetically diverse isolates and reduction of phylogenetic and epidemiological redundancy in the training set can help improve the performance of the source attribution model.

References

1. Bolger AM, Lohse M, Usadel B. Trimmomatic: a flexible trimmer for Illumina sequence data. Bioinformatics. 2014;30:2114–20. PubMed http://dx.doi.org/10.1093/bioinformatics/btu170

2. Bankevich A, Nurk S, Antipov D, Gurevich AA, Dvorkin M, Kulikov AS, et al. SPAdes: a new genome assembly algorithm and its applications to single-cell sequencing. J Comput Biol. 2012;19:455–77. PubMed http://dx.doi.org/10.1089/cmb.2012.0021

3. Gurevich A, Saveliev V, Vyahhi N, Tesler G. QUAST: quality assessment tool for genome assemblies. Bioinformatics. 2013;29:1072–5. PubMed http://dx.doi.org/10.1093/bioinformatics/btt086

4. Kurtz S, Phillippy A, Delcher AL, Smoot M, Shumway M, Antonescu C, et al. Versatile and open software for comparing large genomes. Genome Biol. 2004;5:R12. PubMed http://dx.doi.org/10.1186/gb-2004-5-2-r12

5. Zhou Y, Liang Y, Lynch KH, Dennis JJ, Wishart DS. PHAST: a fast phage search tool. Nucleic Acids Res. 2011 Jul;39(Web Server issue):W347-52.

6. Croucher NJ, Page AJ, Connor TR, Delaney AJ, Keane JA, Bentley SD, et al. Rapid phylogenetic analysis of large samples of recombinant bacterial whole genome sequences using Gubbins. Nucleic Acids Res. 2015;43:e15. PubMed http://dx.doi.org/10.1093/nar/gku1196

7. Didelot X, Wilson DJ. ClonalFrameML: efficient inference of recombination in whole bacterial genomes. PLOS Comput Biol. 2015;11:e1004041. PubMed http://dx.doi.org/10.1371/journal.pcbi.1004041

8. Price MN, Dehal PS, Arkin AP. FastTree 2—approximately maximum-likelihood trees for large alignments. PLoS One. 2010;5:e9490. PubMed http://dx.doi.org/10.1371/journal.pone.0009490

9. Treangen TJ, Ondov BD, Koren S, Phillippy AM. The Harvest suite for rapid core-genome alignment and visualization of thousands of intraspecific microbial genomes. Genome Biol. 2014;15:524. PubMed http://dx.doi.org/10.1186/s13059-014-0524-x

10. Corander J, Marttinen P, Sirén J, Tang J. Enhanced Bayesian modelling in BAPS software for learning genetic structures of populations. BMC Bioinformatics. 2008;9:539. PubMed http://dx.doi.org/10.1186/1471-2105-9-539

11. Li H, Durbin R. Fast and accurate long-read alignment with Burrows-Wheeler transform. Bioinformatics. 2010;26:589–95. PubMed http://dx.doi.org/10.1093/bioinformatics/btp698

12. Garrison E, Marth G. Haplotype-based variant detection from short-read sequencing. arXiv preprint arXiv:12073907. 2012.

13. Guindon S, Dufayard JF, Lefort V, Anisimova M, Hordijk W, Gascuel O. New algorithms and methods to estimate maximum-likelihood phylogenies: assessing the performance of PhyML 3.0. Syst Biol. 2010;59:307–21. PubMed http://dx.doi.org/10.1093/sysbio/syq010

14. Rambaut A, Lam TT, Max Carvalho L, Pybus OG. Exploring the temporal structure of heterochronous sequences using TempEst (formerly Path-O-Gen). Virus Evol. 2016 Jan;2(1):vew007.

15. Drummond AJ, Suchard MA, Xie D, Rambaut A. Bayesian phylogenetics with BEAUti and the BEAST 1.7. Mol Biol Evol. 2012;29:1969–73. PubMed http://dx.doi.org/10.1093/molbev/mss075

16. Suchard MA, Weiss RE, Sinsheimer JS. Bayesian selection of continuous-time Markov chain evolutionary models. Mol Biol Evol. 2001;18:1001–13. PubMed http://dx.doi.org/10.1093/oxfordjournals.molbev.a003872

17. Narzisi G, O'Rawe JA, Iossifov I, Fang H, Lee YH, Wang Z, et al. Accurate de novo and transmitted indel detection in exome-capture data using microassembly. Nat Methods. 2014;11:1033–6. PubMed http://dx.doi.org/10.1038/nmeth.3069

18. Choi Y, Chan AP. PROVEAN web server: a tool to predict the functional effect of amino acid substitutions and indels. Bioinformatics. 2015;31:2745–7. PubMed http://dx.doi.org/10.1093/bioinformatics/btv195

19. Page AJ, Cummins CA, Hunt M, Wong VK, Reuter S, Holden MT, et al. Roary: rapid large-scale prokaryote pan genome analysis. Bioinformatics. 2015;31:3691–3. PubMed http://dx.doi.org/10.1093/bioinformatics/btv421

20. Seemann T. Prokka: rapid prokaryotic genome annotation. Bioinformatics. 2014;30:2068–9. PubMed http://dx.doi.org/10.1093/bioinformatics/btu153

21. Breiman L. Out-of-bag estimation 1996.

22. Okoro CK, Kingsley RA, Connor TR, Harris SR, Parry CM, Al-Mashhadani MN, et al. Intracontinental spread of human invasive *Salmonella* Typhimurium pathovariants in sub-Saharan Africa. Nat Genet. 2012;44:1215–21. PubMed http://dx.doi.org/10.1038/ng.2423

23. Kingsley RA, Kay S, Connor T, Barquist L, Sait L, Holt KE, et al. Genome and transcriptome adaptation accompanying emergence of the definitive type 2 host-restricted *Salmonella enterica* serovar Typhimurium pathovar. MBio. 2013;4:e00565–13. PubMed http://dx.doi.org/10.1128/mBio.00565-13

24. Wu J, Mao X, Cai T, Luo J, Wei L. KOBAS server: a web-based platform for automated annotation and pathway identification. Nucleic Acids Res. 2006;34(Web Server issue):W720-4.

25. Kanehisa M, Goto S. KEGG: kyoto encyclopedia of genes and genomes. Nucleic Acids Res. 2000;28:27–30. PubMed http://dx.doi.org/10.1093/nar/28.1.27

26. Oksanen J, Kindt R, Legendre P, O'Hara B, Stevens MHH. The vegan Package. 2007.

27. Ihaka R, Gentleman R. R: a language for data analysis and graphics. J Comput Graph Stat. 1996;5:299–314.

28. Lupolova N, Dallman TJ, Holden NJ, Gally DL. Patchy promiscuity: machine learning applied to predict the host specificity of *Salmonella enterica* and *Escherichia coli*. Microb Genom. 2017;3:e000135. PubMed http://dx.doi.org/10.1099/mgen.0.000135

29. Kleinheinz KA, Joensen KG, Larsen MV. Applying the ResFinder and VirulenceFinder web-services for easy identification of acquired antibiotic resistance and *E. coli* virulence genes in bacteriophage and prophage nucleotide sequences. Bacteriophage. 2014;4:e27943. PubMed http://dx.doi.org/10.4161/bact.27943

30. Galili T. Hierarchical cluster analysis on famous data sets—enhanced with the dendextend package. 2018

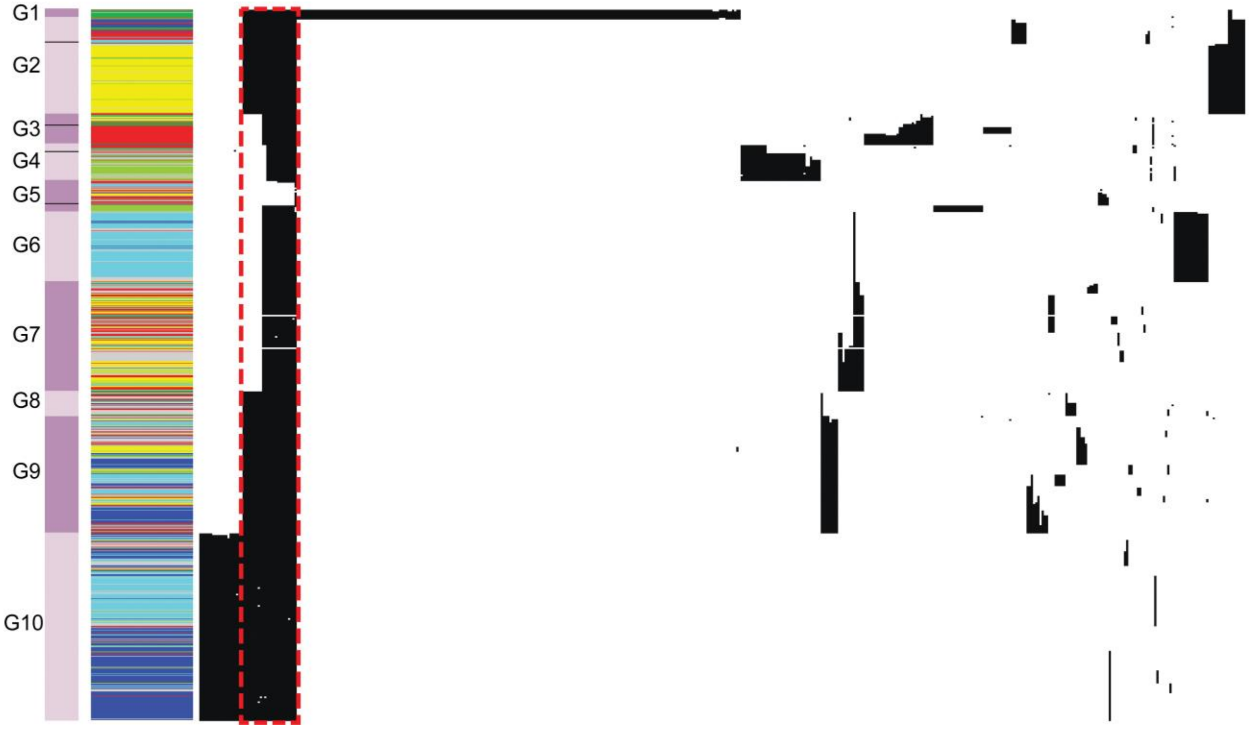

Appendix Figure 1. Distribution of nonsynonymous single-nucleotide polymorphisms and indels among *Salmonella enterica* Typhimurium genomes. *S. enterica* Typhimurium isolates are color coded by source and organized in the same order as they appear in the maximum-likelihood phylogeny (Figure 1, panel A). Cyan, yellow, light green, blue, dark green, red, and grey represents bovine, poultry, wild bird, swine, miscellaneous food, human, and other source, respectively.

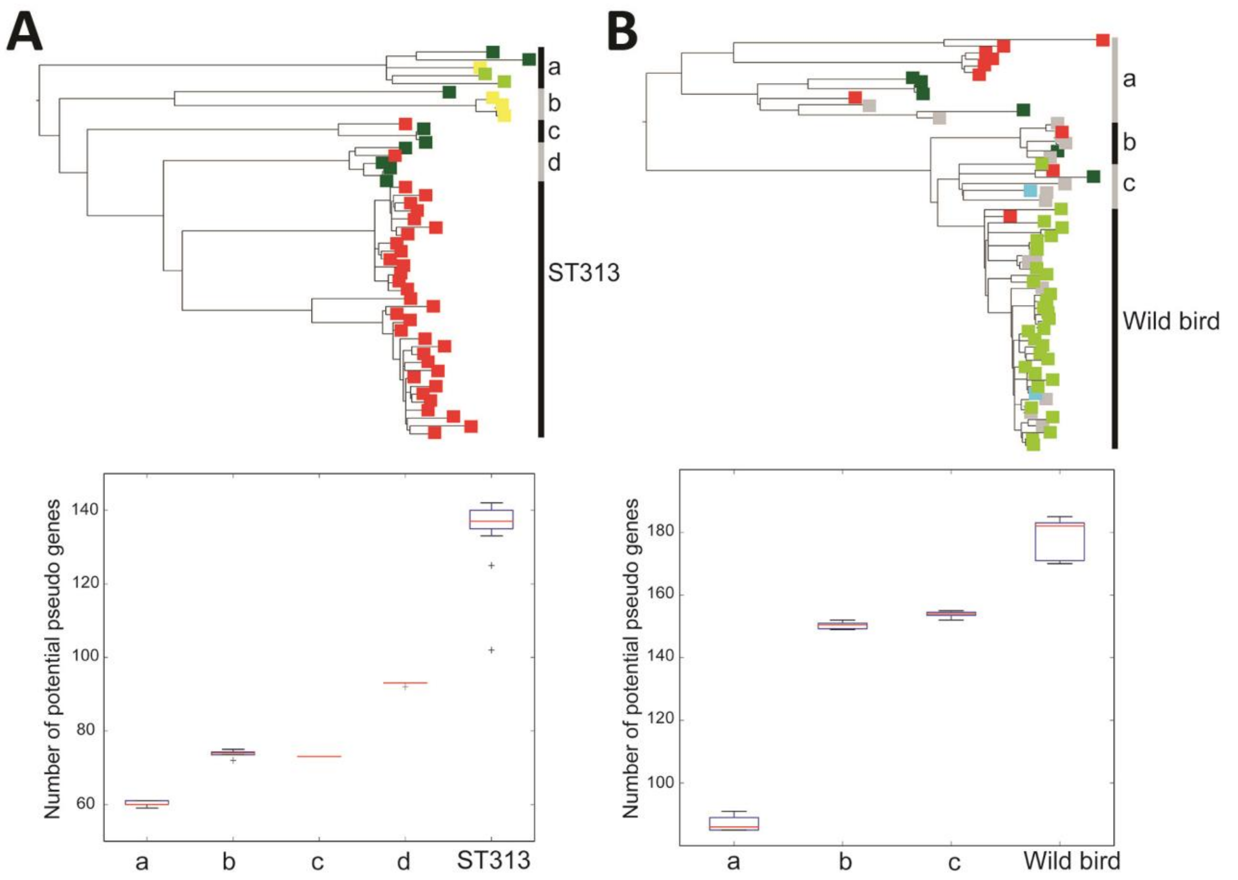

Appendix Figure 2. Examples of elevating accumulation of putative pseudogenes during potential host adaptation. A) The ST313 clade (upper) and putative pseudogene accumulation as it diverged from diverse-source clades (lower). B) Wild bird clade and putative pseudogene accumulation as it diverged from diverse-source clades. Both clades are subtrees of the *S. enterica* Typhimurium phylogeny of 1,267 isolates (Figure 1, panel A). Isolates on the trees are color coded by source (same as Figure 1, panel A): red, humans; dark green, food; yellow, poultry; light green, wild bird; cyan, bovine; blue, swine; grey, other sources.

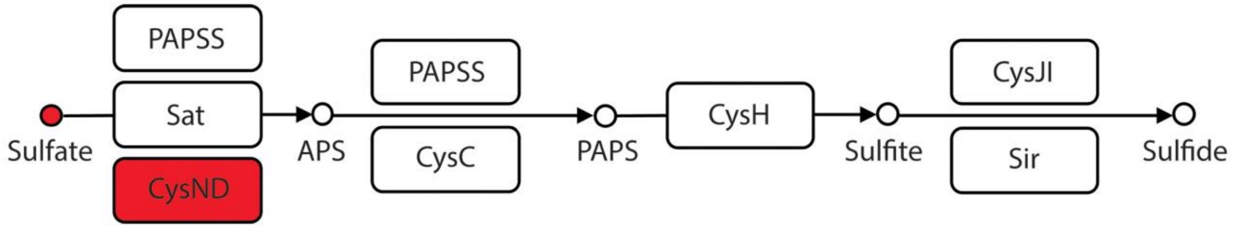

Appendix Figure 3. Assimilatory sulfate reduction pathway potentially disrupted by a nonsynonymous single-nucleotide polymorphism in a sulfate adenylyltransferase subunit gene (*cysN*). PAPSS, 3′-phosphoadenosine 5′-phosphosulfate synthase; Sat, sulfate adenylyltransferase; APS, adenosine-5′-phosphosulfate; PAPS, 3′-phosphoadenosine-5′-phosphosulfate; CysC, adenylylsulfate kinase; CysH, phosphoadenosine phosphosulfate reductase; CysJI, sulphite reductase flavoprotein (CysJ) and haem protein (CysI) subunits; Sir, sulfite reductase.

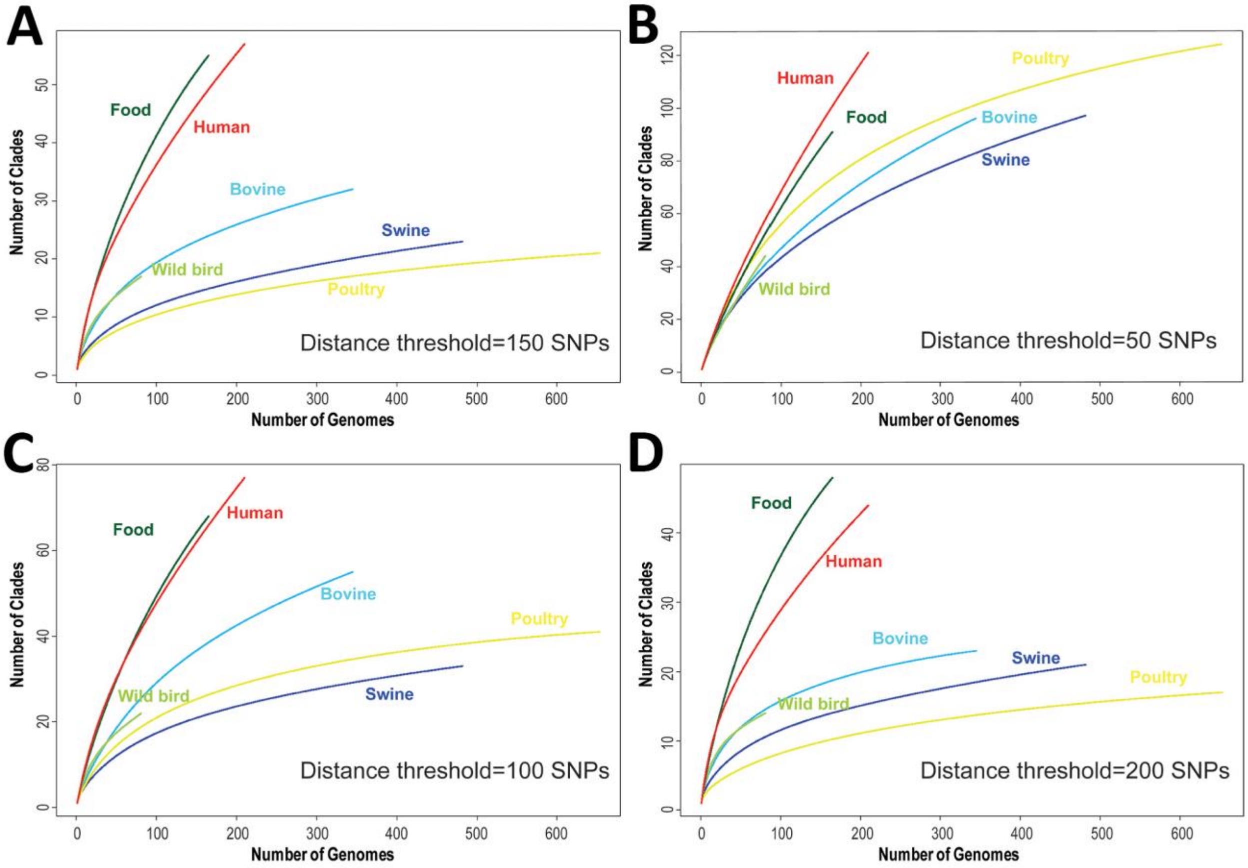

Appendix Figure 4. Rarefaction analysis relative sampling intensities by source. Each panel shows the result by adopting a particular single-nucleotide polymorphism distance threshold for defining phylogenetic clusters.

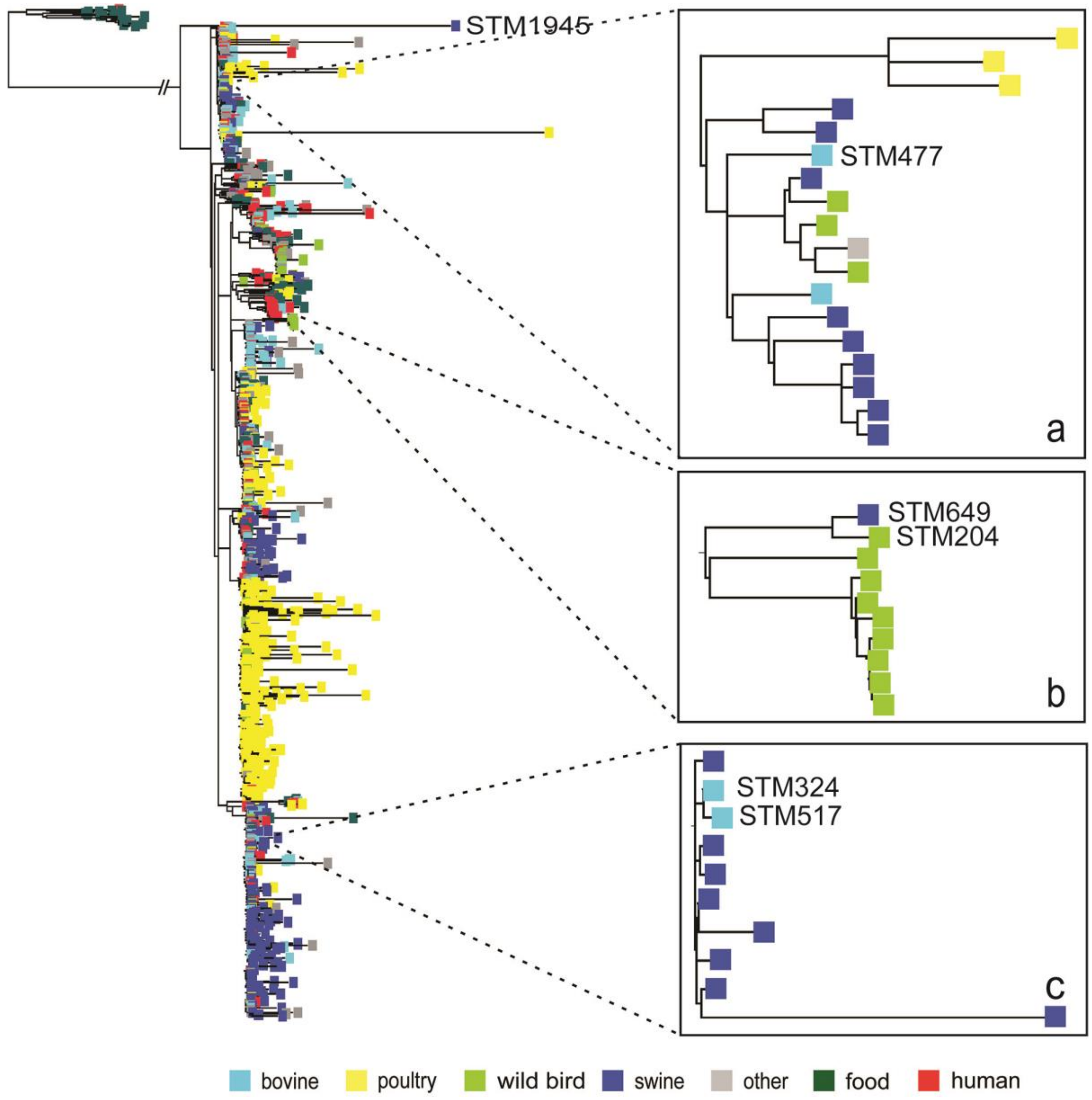

Appendix Figure 5. Examples of *Salmonella enterica* Typhimurium zoonotic source prediction by phylogenetic placement and Random Forest classifier. A total of 1,473 genomes were included in the tree. The original 1,267 *S. enterica* Typhimurium genome dataset for phylogenomic analyses was updated by 1) adding 939 *S. enterica* Typhimurium genomes that became available in GenomeTrakr from September 2015 (initially 1,267 genomes) to January 2017; 2) sequencing another 11 *S. enterica* Typhimurium isolates from 5 outbreaks with confirmed livestock origin in the United States from 2007 to 2013, which, together with 6 livestock isolates from 3 outbreaks in the original data set, led to a total of 8 zoonotic outbreaks for retrospective source attribution; and 3) excluding 744 redundant genomes to minimize biases due to repeated sampling of closely related strains. The genome updates resulted in a modified *S. enterica* Typhimurium collection of 1,473 isolates for source prediction. The 3 panels show the highlighted clades on the phylogenetic tree.

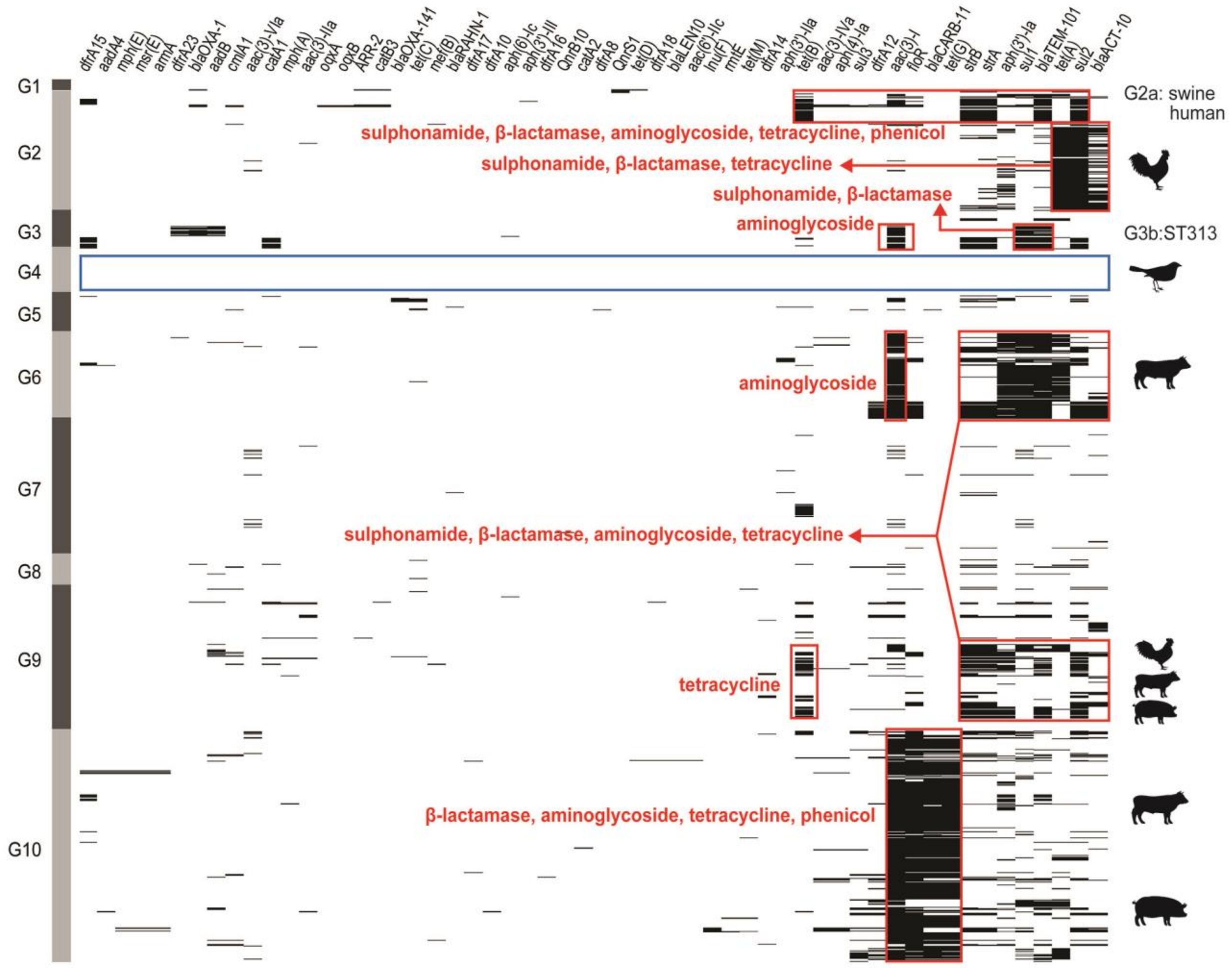

Appendix Figure 6. Distribution of antimicrobial resistance genes among *Salmonella enterica* Typhimurium genomes. Each black line stands for the presence of a specific gene. The vertical order of genomes is the same as that of the phylogenetic tree of 1,267 *S. enterica* Typhimurium genomes. Antimicrobial resistance gens surveyed and major population groups identified were labeled.

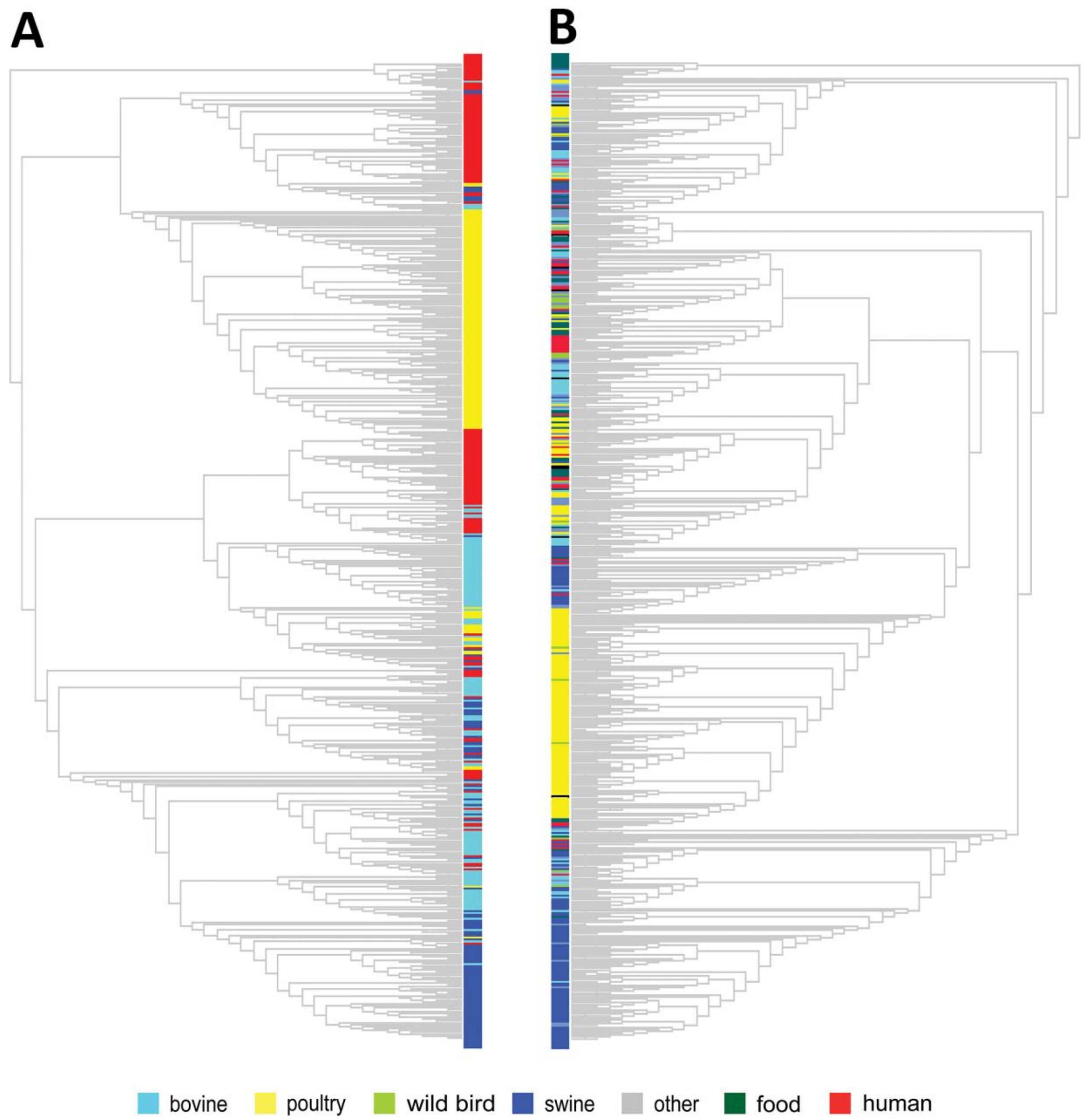

Appendix Figure 7. Comparison of human isolates clonality between the current and a previous study. A) Maximum-likelihood cladogram of *Salmonella enterica* Typhimurium genomes used for classifier training in a previous study based on core genome single-nucleotide polymorphisms. B) Maximum-likelihood cladogram of *S. enterica* Typhimurium genomes used for classifier training in the current study based on core genome single-nucleotide polymorphisms.
